# COVID-19 and menstrual-related disturbances: a Spanish retrospective observational study in formerly menstruating women

**DOI:** 10.3389/fgwh.2024.1393765

**Published:** 2024-07-30

**Authors:** María González, Miriam Al-Adib, Ana B. Rodríguez, Cristina Carrasco

**Affiliations:** ^1^ Grupo de investigación Neuroinmunofisiología y Crononutrición, Departamento de Fisiología, Universidad de Extremadura, Badajoz, Spain; ^2^ Obstetrics & Gynecology Clinics “Miriam Gine”, Badajoz, Spain

**Keywords:** COVID-19, menstruation disturbances, amenorrhea, perimenopause, menorrhagia, women

## Abstract

**Introduction:**

Four years after the start of the pandemic, there is limited evidence on the impact of COVID-19 on the women's health regardless of their reproductive status.

**Objective:**

The aim was to analyze the prevalence and associated factors of menstrual-related disturbances in formerly menstruating women following SARS-CoV-2 infection.

**Study design:**

A retrospective observational study of adult women in Spain was conducted during the month of December 2021 using an online survey (*N* = 17,512). The present analysis includes a subpopulation of SARS-CoV-2-infected and formerly menstruating women (*n* = 72). The collected data included general characteristics, medical history, and specific information on COVID-19. Chi-square and Mann-Whitney *U*-tests were performed. Bivariate logistic regression analysis was then performed to investigate possible associations between the occurrence of menstrual-related disturbances after SARS-CoV-2 infection.

**Results:**

38.8% of participants experienced menstrual-related disturbances following COVID-19. Among these, unexpected vaginal bleeding (20.8%) was the most common event, followed by spotting (11.1%) ( Table 1). Other reported changes were in the length (shorter = 12.5%) and flow (heavier = 30.3%) of menstrual bleeding in comparison to their previous experience. Regression analysis revealed that being a perimenopausal woman [adjusted odds ratio (AOR) 4.721, CI 95%, 1.022–21.796, *p* = 0.047] and having a previous diagnosis of menorrhagia (AOR 5.824 CI 95%, 1.521–22.310, *p* = 0.010) were factors associated with the event.

**Conclusion:**

These findings could help health professionals provide their patients with up-to-date scientific information to empower them to actively manage their reproductive health, especially in societies where menstrual health is still taboo.

## 1 Introduction

Severe acute respiratory syndrome coronavirus 2 (SARS-CoV-2) has been reported to trigger multisystem complications (1, 2). This is due to the ubiquitous expression of the membrane protein angiotensin-converting enzyme 2 (ACE2) (2, 3) and other co-receptors (4), for example in the ovaries, independent of age and ovarian reserve (5). ACE2 has a significant role in the different phases of the menstrual cycle through angiotensin-(1–7) (6, 7). Changes in this molecular pathway and other neuroendocrine axes due to COVID-19 may result in menstrual cycle irregularities (4, 8) as well as the corresponding immune response (8). Thus, it would be expected that SARS-CoV-2 could temporarily or even permanently impair female fertility. Unfortunately, our knowledge of the basic uterine and menstrual physiology is insufficient to understand more complex processes of this kind.

Previous studies have linked viral infections to changes in women's reproductive health (9–11). However, there are conflicting results regarding SARS-CoV-2 infection in menstruating women (12–14). In addition, the prevalence of menstrual-related disturbances following COVID-19 (MRD-COVID19) in formerly menstruating women (FMW)—that is, those who were not menstruating at the time of infection for various reasons−remains unknown. The medical term for this is “secondary amenorrhea”. It is characterized by missing three menstrual periods in a row or not having periods for at least 6 months after menstruating normally. Common causes include pregnancy, breastfeeding, menopause, the use of contraceptives, and gynecological conditions (15, 16), most of which are exclusion criteria in similar studies. For this reason, the aim was to analyze the health factors that might be associated with the occurrence of MRD-COVID19 in FMW at the time of infection.

## 2 Method

### 2.1 Experimental design

A retrospective observational study was conducted among adult Spanish women using an online survey (Microsoft Forms®, Microsoft Corporation, Washington, USA). The study was conducted in accordance with the Declaration of Helsinki and approved by the Institutional Review Board of University of Extremadura (ref. 180/2021).

### 2.2 Recruitment, data collection and participants

**Figure 1:**
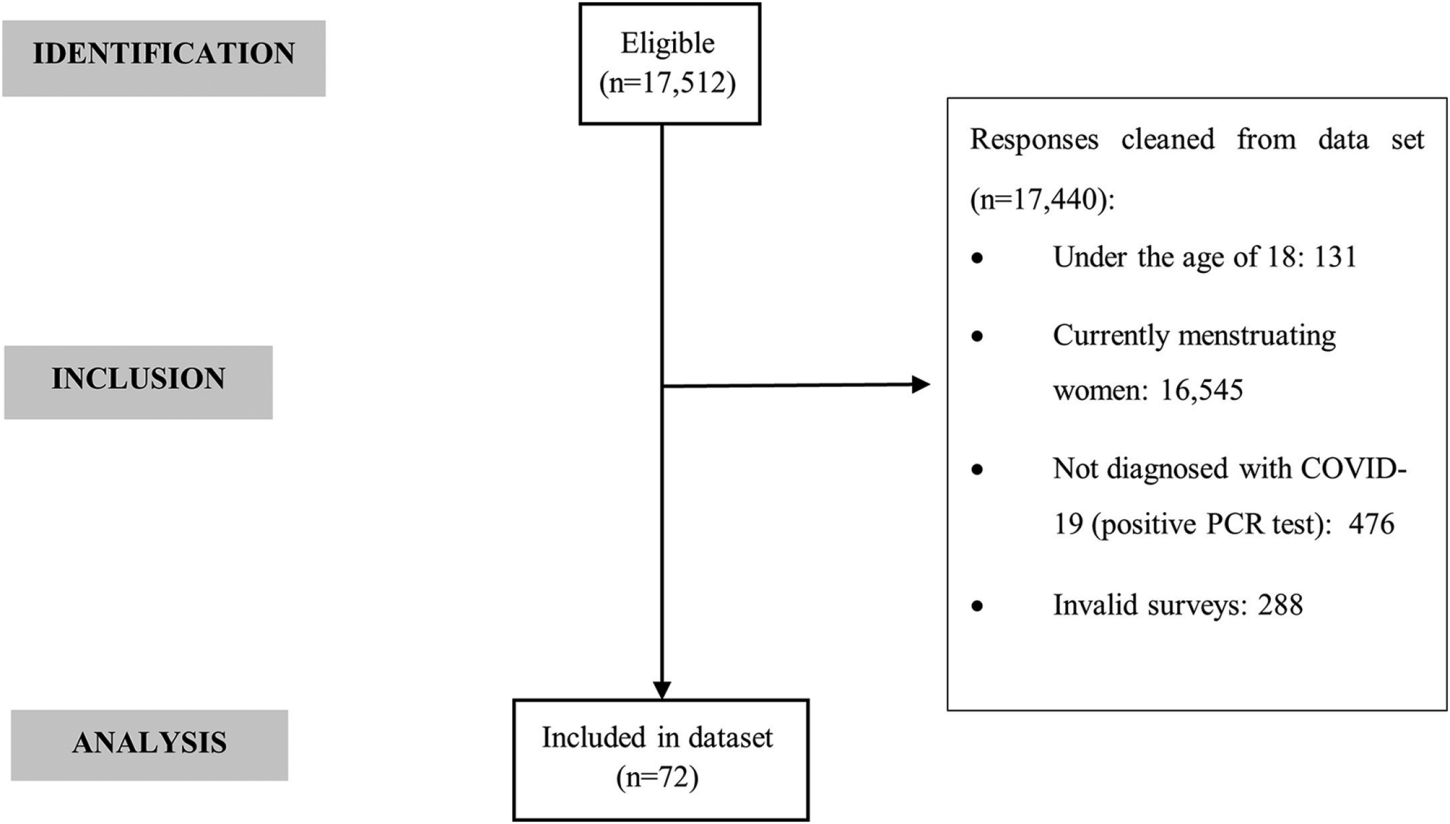
STROBE (strengthening the reporting of observational studies in epidemiology) flow diagram.

The online survey was published in Spain in December 2021 through social networks, using the snowball method. Informed consent was only obtained from those who agreed to be contacted by the research group by email for additional data collection. A total of 17,512 women were recruited within 15 days, regardless of their menstrual status. These results describe a subgroup analysis (*n* = 72 FMW, Figure 1) of this larger study; thus, the inclusion criteria were women: (i) over 18 years of age, (ii) with secondary amenorrhea of any cause prior to COVID-19 diagnosis, and (iii) with a diagnosis of COVID-19 (positive PCR test). The research sample excluded women who were currently menstruating at the time of infection or had not been diagnosed with the disease. The minimum representative sample size of the total population of Spanish women of reproductive age (*N* = 8,431,595) was calculated, considering an estimated prevalence of secondary amenorrhea of 4.0% (17). The following parameters were considered: statistical power 80.0%, alpha = 0.5 and effect size 1. This resulted in a sample size of 60 formerly menstruating women.

### 2.3 Survey information

A customized questionnaire was designed based on the survey conducted by Lee et al. (18) in April 2021. It consisted of 56 multiple-choice and text-entry questions divided into 6 sections. Participants were asked about (1) the general characteristics of their menstrual cycles—or its absence and the cause −, (2) SARS-CoV-2 infection, (3) COVID-19 vaccine, (4) menstrual experiences both after the SARS-CoV-2 infection and COVID-19 vaccination in comparison with the expected period symptoms−e.g., shorter/longer/same, heavier/lighter/same…−, (5) other menstrual changes−e.g., spotting, breast pain, hot flashes, premenstrual syndrome and abnormal bleeding −, (6) time between infection/vaccine and menstrual changes, (7) duration of the menstrual changes, (8) adverse events from each dose of the vaccine, (9) reproductive history, (10) medical history, and (11) demographics. The survey took 20–25 min to complete.

### 2.4 Statistical analysis

Participants were categorized according to the occurrence (MRD subgroup) or non-occurrence (n-MRD subgroup) of MRD-COVID19. Values were expressed as median and interquartile range, and as number of participants and frequency (%) for qualitative and quantitative variables, respectively. Chi-square and Mann-Whitney *U*-tests were performed. Bivariate logistic regression analysis was then performed. Results were presented as adjusted odds ratios (AORs) with 95% confidence intervals (CI). The above analyses were performed using the Statistical Package for Social Sciences (SPSS v.25, IBM, New York, USA) for Windows. Statistical significance was set at *p* ≤ 0.05.

## 3 Results

56.9% of the FMW [*N* = 72, median age 40.0 (33.0–51.8) years] had normal weight (median BMI value 24.0 (21.6–27.8), 19.4% had autoimmune diseases, and 27.1% had other clinical conditions. The most common cause of secondary amenorrhea in the study population was contraceptive use (31.9%), followed by postmenopause (22.2%). A total of 52.8% reported having been diagnosed with a gynecological disease, mainly menorrhagia (19.4%) (Supplementary Material S1).

38.8% of participants experienced MRD-COVID19. Among these, unexpected vaginal bleeding (20.8%) was the most common, followed by spotting (11.1%) (Table 1). Other reported changes were in the length (shorter = 12.5%) and flow (heavier = 30.3%) of menstrual bleeding in comparison to their previous experience. When asked about the time between the SARS-CoV-2 infection and the onset of menstrual bleeding, a 20.8% of the FMW answered “more than 14 days”; in 53.3% of cases, it was unexpected compared to the expected period date. Additionally, 27.8% reported that these symptoms had lasted “to date.”

**Table 1:** COVID-19 and the occurrence of MRD in the study population (formerly menstruating women, *N* = 72).

Variable	Category	Total (*N* = 72)
Date of COVID-19 diagnosis (pandemic waves)	1st- 2nd waves	38 (52.8)
	3rd- 5th waves	34 (47.2)
Hospitalization	Yes	2 (2.8)
	No	70 (97.2)
Types of MRD-COVID19	Unexpected vaginal bleeding	15 (20.8)
	Spotting	8 (11.1)
	Shorter/longer menstrual cycle	4 (5.6)
	None	45 (62.5)
Length of menstrual bleeding	Shorter	9 (12.5)
	Unchanged	16 (22.2)
	Longer	8 (11.1)
Flow of menstrual bleeding	Not applicable	39 (54.2)
	Lighter	7 (21.2)
	Unchanged	16 (48.5)
	Heavier	10 (30.3)
Time between SARS-CoV-2 infection and period/abnormal bleeding	I was menstruating when I got infected	1 (1.4)
	After 1–14 days	14 (19.4)
	After more than 14 days	15 (20.8)
	Not applicable	42 (58.3)
Coincidence with period date	Yes	9 (30.0)
	No	16 (53.3)
	Not applicable	5 (16.7)
Premenstrual syndrome		
N° symptoms	2 or more symptoms	14 (56.0)
	None	6 (24.0)
Types	Fluid retention	2 (8.0)
	Pain	1 (4.0)
	Other	2 (2.8)
Duration of MRD-COVID19	1–6 months	11 (15.4)
	6–12 months	3 (4.2)
	To date	20 (27.8)
	Not applicable	38 (52.8)

Values are expressed as: *n* (%).

BMI, body mass index; IUD, intrauterine device; MRD-COVID19, menstrual-related disturbances following COVID-19.

Comparative analysis (Table 2) showed significant differences between subgroups with respect to factors such as perimenopause, menorrhagia, and pandemic waves. Binary logistic regression (Table 3) confirmed that being a perimenopausal woman (AOR 4.721, CI 95%, 1.022–21.796, *p* = 0.047) and having a previous diagnosis of menorrhagia (AOR 5.824 CI 95%, 1.521–22.310, *p* = 0.010) were associated with MRD-COVID19 in FMW.

**Table 2:** Differences in the study variables according to the occurrence or not of MRD-COVID19 in the study population (formerly menstruating women, *N* = 72).

Variable		Category	Subgroup		X^2^	*p*-value
			nMRD-COVID19 (*n* = 44)	MRD-COVID19 (*n* = 28)		
Age (years)^a^		–	37.5 (33.0–53.5)	44.0 (31.5–50.8)	–	0.954
BMI^a,b^		–	24.1 (21.8–27.4)	23.9 (22.2–28.5)	–	0.768
		Underweight	3 (6.8)	2 (7.1)	2.290	0.683
		Normal weight	27 (61.4)	14 (50.0)		
		Pre-obesity/overweight	6 (13.6)	7 (25.0)		
		Obesity	8 (18.2)	5 (17.9)		
Medical history^b^						
Autoimmune diseases	Diagnosis	Yes	9 (20.5)	5 (17.9)	0.074	0.786
		No	35 (79.5)	23 (82.1)		
Other clinical conditions	Diagnosis	Yes	13 (29.5)	6 (21.4)	0.346	0.557
		No	31 (70.5)	20 (71.4)		
Allergies		–	31 (70.5)	13 (29.5)	2.114	0.146
Gynaecological history^a,b^						
Age 1st menstruation		–	13.0 (12.0–14.0)	13.0 (12.0–13.0)	–	0.921
Contraceptives	Time of use	<10 years	12 (27.3)	11 (39.3)	1.810	0.405
		>10 years	3 (6.8)	3 (10.7)		
	Types	None	29 (65.9)	14 (50.0)	2.511	0.285
		Hormonal	11 (25.0)	12 (42.9)		
		IUD (nonhormonal)	4 (9.1)	2 (7.1)		
Reproduction	Have you ever been pregnant?	Yes	27 (61.4)	17 (60.7)	0.003	0.956
		No	17 (38.6)	11 (39.3)		
	N° pregnancies	0–2	37 (84.1)	23 (82.1)	0.047	0.290
		> 2	7 (15.9)	5 (17.9)		
	N° children	0–2	40 (90.9)	28 (100.0)	2.695	0.101
		> 2	4 (9.1)	0 (0.0)		
2° amenorrhea	Causes	Perimenopause	3 (6.8)	8 (28.6)*	6.809	0.033
		Postmenopause	12 (27.3)	4 (14.3)		
		Other	29 (65.9)	16 (57.1)		
Diseases	Types	Endometriosis	3 (6.8)	5 (17.9)	2.111	0.146
		Fibroids	2 (4.5)	2 (7.1)	0.220	0.639
		Other	5 (11.4)	3 (10.7)	0.007	0.932
		Menorrhagia	4 (5.6)	10 (35.7)*	7.743	0.005
		PCOS	8 (18.2)	5 (17.9)	0.001	0.972
COVID-19						
Date of COVID-19 diagnosis (pandemic waves)	1st wave		15 (34.1)	6 (21.4)	10.378	0.035
	2nd wave		9 (20.5)	8 (28.6)		
	3rd wave		10 (22.7)	2 (7.1)		
	4th wave		1 (2.3)	6 (21.4)*		
	5th wave		9 (20.5)	6 (21.4)		
Hospitalization	Yes		0 (0.0)	2 (7.1)	3.233	0.072
	No		44 (100.0)	26 (92.9)		

c *p < 0.05 vs. subgroup nMRD-COVID19.

BMI, body mass index; IUD, intrauterine device; MRD-COVID19: menstrual-related disturbances subgroup; nMRD-COVID19, non-menstrual-related disturbances subgroup; PCOS, polycystic ovary syndrome; HPV, human papillomavirus.

^a^median (interquartile range); ^b^n (%).

**Table 3:** Factors associated with the occurrence of nMRD-COVID19 (formerly menstruating women, *N* = 72): binary logistic regression.

Variable	Category	Subgroup MRD (*n* = 28)	*p*-value
		AOR (CI 95%)	
2° amenorrhea	Perimenopause	4.721 (1.022–21.796)	0.047
	Postmenopause	0.551 (0.138–2.195)	0.398
	Other	1	
Menorrhagia	Yes	5.824 (1.521–22.310)	0.010
	No	1	
Pandemic wave	3rd-5th	1.161 (0.402–3.358)	0.782
	1st-2nd	1	

Reference group: Do not experience MRD-COVID19.

AOR adjusted odd ratio; CI 95%, confidence interval.

## 4 Discussion

Although it is now well established that COVID-19 exhibits sex differences due to several biological factors, very few studies have analyzed the impact of this disease on the female reproductive system during the different stages of a woman's life. Based on the limited scientific evidence available, menstrual changes might affect 16%–25% of women of childbearing age infected with SARS-CoV-2 (12, 19, 20). The most commonly reported disturbances are worsened premenstrual syndrome, irregular and infrequent menstruation (20), and decreased menstrual volume (12, 19). In addition, other authors have reported a high prevalence of post-COVID-19 menstrual/period issues in women aged 30–60 years (21–23), including cycle length, menstrual flow and menses duration (23). One of the most notable findings of our study is that women who were not menstruating at the time of infection due mainly to contraceptive use or perimenopause/menopause, also experience unexpected menstrual cycle-related events, such as vaginal bleeding, spotting or changes in the length/flow of menstrual bleeding. Overall, this evidence suggests that there are differences in the prevalence and characteristics of the menstrual changes between young and middle-aged women. which need to be confirmed by further research. Indeed, the regression analysis showed that being a perimenopausal woman was a factor associated with MRD-COVID-19. Furthermore, it cannot exclude that some women may also experience long-term menstrual changes (12, 19, 21).

As Khan et al. (20) point out, the menstrual cycle involves complex interactions and can therefore be influenced by a variety of factors, including viral infections (9–11, 24). Therefore, potential direct and indirect effects of SARS-CoV-2 on the occurrence of menstrual changes need to be considered. Systemically, the damage is thought to be mediated by a direct viral role, pro-inflammatory immune responses, imbalances in physiological systems −e.g., the renin-angiotensin-aldosterone system and the ACE2/angiotensin-(1–7)/mitochondrial angiotensin axis, and the HPG and HPA axes −, and the downregulation of ACE2 expression (2, 25). This adverse context could be exacerbated by local direct effects in the female reproductive system with undefined consequences for menstrual physiology (13, 26). It should be noted that estrogens are well known to act in a coordinated manner with the immune system and metabolism (2, 18, 26). Estradiol has been suggested to play a protective role in COVID-19 through several pathways (26, 27). Therefore, the abnormal sex hormone secretion resulting from the SARS-CoV-2 infection may also alter the immune-neuro-endocrine network. However, the evidence remains inconclusive (12, 13). This raises the question of whether the prevalence and the characteristics of menstrual disturbances may be subject to the fluctuations in hormone levels at different stages not only of the menstrual cycle, but also of a woman's life. This assumption may also explain the reported differences in primary COVID-19 outcomes according to the menstrual status and contraceptive use (28, 29). Considering that perimenopause is a transitional phase mainly characterized by lower circulating levels of estradiol, it is suggested that the resulting dampened immune response, the downregulation of autophagy and the altered expression of ACE2 and other co-receptors such as transmembrane protease serine subtype 2, dipeptidyl peptidase-4 and furin (26, 27) may underlie the increased risk of experiencing this unexpected event. Conversely, high levels of estrogen, and consequently increased estrogen receptor signaling, may prevent further respiratory complications in SARS-CoV-2-infected pregnant women (29, 30). For this reason, estrogen supplementation has been proposed as a therapeutic approach to reduce the severity of the COVID-19 (30, 31). For Mateus et al. (32), not only estrogen, but sex hormones as a whole could justify the differences between sexes and age rates, which makes sense given the opposite effects of testosterone on immune response and virus clearance compared to estradiol (26, 27).

On the other hand, other concurrent factors in the prevalence of MRD-COVID19 in FMW should not be ignored, including comorbidity (12, 30). Severe acute illnesses can affect ACE2 levels (33) and HPG axis, leading to reduced levels of progesterone and estrogens (34). Surprisingly, the prevalence of autoimmune diseases or allergies did not differ between subgroups in our study. Only menorrhagia was associated with the MRD-COVID19; in this case, the imbalance between estrogens and progesterone levels may underlie this unexpected event (35, 36). Overall, it is worth considering whether the endocrine disorder observed in female COVID-19 patients is a consequence of systemic rather than local effects, such as nervous system injury (37) and pituitary dysfunction (13). However, there are conflicting results on SARS-CoV-2 neuroinvasion (37). Stress-associated, neuroendocrine-immune mechanisms should also be considered (18, 19, 22, 38), particularly during hospitalization (7), as well as genetics, socio-demographics, culture, and lifestyle factors (19, 21, 32, 39). Finally, the distinct levels of infectivity and transmissibility of the SARS-CoV-2 variants (40) could also have influenced this event. However, we could not prove this hypothesis and only found significant differences for the fourth wave in Spain (alpha variant or B.1.1.7).

This study is one of the few worldwide to focus on the impact of COVID-19 in FMW, demonstrating that women may experience menstrual-related disturbances regardless of their reproductive status at the time of SARS-CoV-2 infection. Some of the limitations include the small sample size, risk of recall bias or self-selection and the experimental design, as well as the lack of knowledge about the existence and importance of certain covariates −and the potential confounding effects −. In addition, the heterogeneity of the study population makes it difficult to fully understand the phenomenon due to the different nature of the underlying factors of secondary amenorrhea. However, we consider that this is a starting point for future research on the impact on viral infection in this subpopulation. Our findings here may not be applicable to other countries than Spain. A longitudinal and multinational study could help to establish the cause-effect relationship and to determine more precisely the factors associated with the occurrence of MRD-COVID19.

In conclusion, menstrual disturbances may be more likely to occur in perimenopausal FMW after COVID-19. The lack of knowledge about various aspects of women's health continues to lead to underestimation or direct ignorance of this phenomenon. These findings could help healthcare professionals to provide their patients with scientifically up-to-date information to enable them to make informed decisions about their reproductive choices.

## Data Availability

The data presented in this study are available upon request from the corresponding author. The data are not publicly available due to ethical restrictions.
