# Supplementary material for: COVID-19 and menstrual-related disturbances: a Spanish retrospective observational study in formerly menstruating women
Source: Front Glob Womens Health. 2024 Jul 30;5:1393765. doi: 10.3389/fgwh.2024.1393765 (PMC11319254; doi:10.3389/fgwh.2024.1393765)
Supplement: Supplementary file 1 [file Table1.docx]

Supplementary Material

**Appendix 1.** Anthropometric characteristics and medical history of the study population (formerly menstruating women, N=72).

| **Variable** |  | **Category** | **Total  (N=72)** |
| --- | --- | --- | --- |
| Age (years) ^a^ |  | - | 40.0 (33.0-51.8) |
| BMI ^a, b^ |  | - | 24.0 (21.6-27.8) |
|  |  | Underweight | 5 (6.9) |
|  |  | Normal weight | 41 (56.9) |
|  |  | Pre-obesity/overweight | 13 (18.1) |
|  |  | Obesity | 13 (18.1) |
| Medical history ^b^ |  |  |  |
| Autoimmune diseases | Diagnosis | Yes | 14 (19.4) |
|  |  | No | 58 (19.4) |
|  | Comorbidity | Yes | 1 (7.1) |
|  |  | No | 13 (92.9) |
|  | Types | Thyroid | 6 (8.3) |
|  |  | Gastrointestinal | 3 (4.2) |
|  |  | Other | 3 (4.2) |
|  |  | Dermatological | 2 (2.8) |
|  |  | Rheumatic/articular | 2 (2.8) |
| Other clinical conditions | Diagnosis | Yes | 19 (27.1) |
|  |  | No | 51 (72.9) |
|  | Comorbidity | Yes | 5 (23.8) |
|  |  | No | 16 (76.2) |
|  | Types | Other | 6 (8.3) |
|  |  | Gynaecological | 4 (5.6) |
|  |  | Cancer | 3 (4.2) |
|  |  | HPV | 3 (4.2) |
|  |  | Cardiovascular | 2 (2.8) |
|  |  | Gastrointestinal | 2 (2.8) |
|  |  | Neurological/mental | 1 (1.4) |
|  |  | Respiratory | 1 (1.4) |
|  |  | Rheumatic/articular | 1 (1.4) |
|  |  | Thyroid | 1 (1.4) |
| Allergies | Diagnosis | Yes | 26 (36.1) |
|  |  | No | 46 (63.9) |
| Gynaecological history ^a, b^ |  |  |  |
| Age 1st  menstruation |  | - | 13.0 (12.0-14.0) |
|  |  |  |  |
| Secondary  amenorrhea | Causes | Contraceptives | 23 (31.9) |
|  |  | Postmenopause | 16 (22.2) |
|  |  | Other | 14 (19.4) |
|  |  | Perimenopause | 11 (15.3) |
|  |  | Breastfeeding | 8 (11.1) |
| Current/ past use of contraceptives | Time of use | <10 years | 23 (31.9) |
|  |  | >10 years | 6 (8.3) |
|  | Types | None | 43 (59.7) |
|  |  | Hormonal | 23 (31.9) |
|  |  | IUD (nonhormonal) | 6 (8.3) |
| Reproduction | Have you ever been pregnant? | Yes | 44 (61.1) |
|  |  | No | 28 (38.9) |
|  | Nº pregnancies | 0-2 | 60 (83.3) |
|  |  | >2 | 16 (16.7) |
|  | Nº children | 0-2 | 68 (94.4) |
|  |  | >2 | 4 (5.6) |
| Diseases | Diagnosis | Yes | 38 (52.8) |
|  |  | No | 34 (47.2) |
|  | Comorbidity | Yes | 7 (18.4) |
|  |  | No | 34 (47.2) |
|  | Types | PCOS | 13 (18.1) |
|  |  | Endometriosis | 8 (11.1) |
|  |  | Menorrhagia | 14 (19.4) |
|  |  | Other | 8 (11.1) |
|  |  | Fibroids | 4 (5.6) |

Values are expressed as: ^a^ median (interquartile range); ^b^ n (%).

Abbreviations: BMI, Body Mass Index; IUD, Intrauterine Device; PCOS, Polycystic Ovary Syndrome; HPV, Human Papillomavirus.
